# Supplementary material for: Change in unemployment by social vulnerability among United States counties with rapid increases in COVID-19 incidence—July 1–October 31, 2020
Source: PLoS One. 2022 Apr 20;17(4):e0265888. doi: 10.1371/journal.pone.0265888 (PMC9020703; doi:10.1371/journal.pone.0265888)
Supplement: S1 Fig — US rapid riser county map. The map was generated using SAS 9.4; basemap: UNITED STATES—COUNTIES: Copyright(C) 1996. SAS Institute Inc. Created and last modified 06/25/2015. The data displayed in the map are available at: Centers for Disease Control and Prevention/ Agency for Toxic Substances and Disease Registry/ Geospatial Research, Analysis, and Services Program. CDC/ATSDR Social Vulnerability Index [2018] Database [US]. https://www.atsdr.cdc.gov/placeandhealth/svi/data_documentation_download.html Accessed on [January 10th, 2022]. https://usafacts.org/visualizations/coronavirus-covid-19-spread-map. (DOCX) [file pone.0265888.s001.docx]

**S1 Fig: COVID-19 Rapid Riser Counties^†^ by Social Vulnerability Index (SVI) Quartile--- United States, July 1-October 31, 2020**


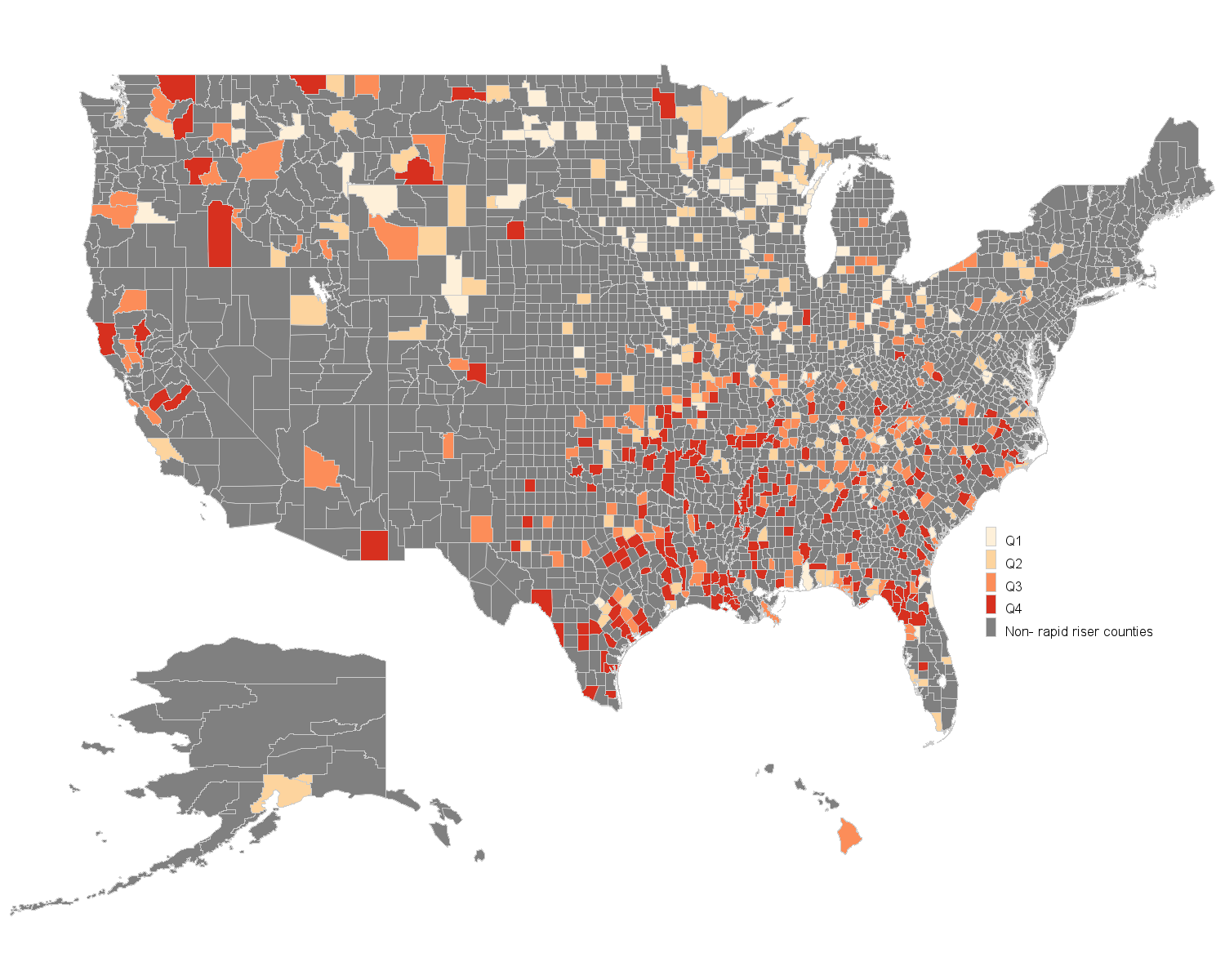


^†^Rapid riser counties were defined as those that met all of the following criteria: 1) >100 new cases in recent week, 2) >0% change in the 7-day incidence, 3) >-60% change in the 3-day incidence, and 4) a 7-day incidence / 30-day incidence ratio >0.31. In addition, rapid riser counties met one or both of the following triggering criteria: 1) >60% change in 3-day incidence, or 2) >60% change in 7-day incidence. For this analysis, we categorized a county as a rapid riser if the county met the standardized daily rapid riser criteria on at least three days in the week.

Note: The map was generated using SAS 9.4; basemap: UNITED STATES - COUNTIES: Copyright(C) 1996. SAS Institute Inc. Created and last modified 06/25/2015. The data displayed in the map are available at: Centers for Disease Control and Prevention/ Agency for Toxic Substances and Disease Registry/ Geospatial Research, Analysis, and Services Program. CDC/ATSDR Social Vulnerability Index [2018] Database [US]. https://www.atsdr.cdc.gov/placeandhealth/svi/data_documentation_download.html. Accessed on [January 10th, 2022].<https://usafacts.org/visualizations/coronavirus-covid-19-spread-map>
