# Supplementary material for: Change in unemployment by social vulnerability among United States counties with rapid increases in COVID-19 incidence—July 1–October 31, 2020
Source: PLoS One. 2022 Apr 20;17(4):e0265888. doi: 10.1371/journal.pone.0265888 (PMC9020703; doi:10.1371/journal.pone.0265888)
Supplement: S1 Table — Sensitivity analysis table. (DOCX) [file pone.0265888.s002.docx]

**S1 Table: Unemployment Rate Gap Changes (β) by Social Vulnerability Index (SVI), Overall and by SVI Theme, among Rapid Riser Counties^†^ (N = 585) Before and After**^¶^ **to a Rapid Rise in COVID-19 Incidence --- United States**

|  | **Unemployment Rate Gap Change** | |
| --- | --- | --- |
| **Social Vulnerability Index (SVI) Quartile, by SVI Theme** | **β^§^** | **(95% CI)** |
| **Overall SVI^††^** |  |  |
| Q1 (lowest vulnerability) | Reference |  |
| Q2 | -0.11 | (-0.36, 0.14) |
| Q3 | -0.11 | (-0.37, 0.15) |
| Q4 (highest vulnerability) | **0.36**** | (0.10, 0.62) |
|  |  |  |
| **SVI related to socioeconomic status**^¶¶^ |  |  |
| Q1(lowest vulnerability) | Reference |  |
| Q2 | 0.09 | (-0.16, 0.35) |
| Q3 | **0.26*** | (0.01, 0.52) |
| Q4 (highest vulnerability) | **0.52**** | (0.26, 0.78) |
|  |  |  |
| **SVI related to household composition & disability** |  |  |
| Q1(lowest vulnerability) | Reference |  |
| Q2 | -0.06 | (-0.31, 0.18) |
| Q3 | 0.03 | (-0.24, 0.29) |
| Q4 (highest vulnerability) | 0.15 | (-0.11, 0.41) |
|  |  |  |
| **SVI related to minority status & language** |  |  |
| Q1(lowest vulnerability) | Reference |  |
| Q2 | 0.24 | (-0.02,0.51) |
| Q3 | **0.41**** | (0.14,0.68) |
| Q4 (highest vulnerability) | **0.38*** | (0.06,0.70) |
|  |  |  |
| **SVI related to housing type & transportation** |  |  |
| Q1(lowest vulnerability) | Reference |  |
| Q2 | -0.16 | (-0.46, 0.13) |
| Q3 | 0.01 | (-0.27, 0.29) |
| Q4 (highest vulnerability) | 0.06 | (-0.23, 0.34) |
|  |  |  |
| Boldface indicates statistical significance (**p*<0.05, ***p*<0.01). | | |

^†^Rapid riser counties were defined as those that met all of the following criteria: 1) >100 new cases in recent week, 2) >0% change in the 7-day incidence, 3) >-60% change in the 3-day incidence, and 4) a 7-day incidence / 30-day incidence ratio >0.31. In addition, rapid riser counties met one or both of the following triggering criteria: 1) >60% change in 3-day incidence, or 2) >60% change in 7-day incidence. For this analysis, we categorized a county as a rapid riser if the county met the standardized daily rapid riser criteria on at least three days in the week.

^¶^ Before rapid rise is defined as the 2 months preceding the rapid rise index month. After rapid rise is defined as the rapid rise index month plus the following month.

^§^ The coefficient of regression.

**^††^** The unemployment was excluded from the overall SVI.

^¶¶^ The unemployment was excluded from the SVI social economic status theme.
