# Supplementary material for: Change in unemployment by social vulnerability among United States counties with rapid increases in COVID-19 incidence—July 1–October 31, 2020
Source: PLoS One. 2022 Apr 20;17(4):e0265888. doi: 10.1371/journal.pone.0265888 (PMC9020703; doi:10.1371/journal.pone.0265888)
Supplement: Attachment — Submitted filename: PLOS1 Article.docx [file pone.0265888.s003.docx]

I thank the authors for an interesting, important, and timely article. I have included here a few comments and questions that I think will help improve the article.

Introduction

1. Might be worth updating the introduction sentence based on more recent COVID numbers (could update this closer to publication timeline)
2. In the third sentence on the way that a community can cope with a disaster, it’s not just unemployment – perhaps rephrase to talk about other work-related factors: underemployment, distribution of essential and/or public facing jobs (which in turn put different groups of people at higher risk for COVID), as well as unemployment.
3. Unemployment is also tied to negative health impacts. Perhaps rephrase the first sentence of the second paragraph to get at the direct health impacts of COVID as well as the indirect (which impact employment, which in turn impact health 🡪 e.g. someone has to leave their work, and their work is how they received health insurance so now they are uninsured and management of chronic health conditions deteriorates, as well as the impact of being unemployed on stress levels which in turn impacts health)
4. It would help to say a little more about the SVI in the introduction. Perhaps a few additional references of how it’s been used?

Methods

5. You use SVI data from 2018 – is this the most recently available? Can you comment (or maybe you do in the discussion) about how this may differ from the situation in 2020? (I see this commented on in the limitations in the discussion, but perhaps acknowledging this time gap in the methods too?
6. You mention that there were 3.141 US counties with complete data – how many counties total in the US? What is the %complete?

Discussion

7. Can you comment on you might hypothesize how things might differ in other periods? For example, during the winter peak, initial peak, or once the vaccine became widely available?
8. One thing that I don’t think is reflected in the SVI is that not all jobs are created equal. That is, especially in times of COVID, some jobs put people at increased risk due to the nature of the work. For example, public facing jobs, essential jobs – grocery stores, bus drivers, etc. – these people are deemed working, but face increased risk compared to people that can work from home. We see racial and ethnic inequities based on who works in these jobs (see for example <https://www.mass.gov/doc/characterizing-ma-workers-in-select-covid-19-essential-services-food-stores-and-urban-transit/download>. Perhaps worth mentioning something about this limitation of the SVI?
